# Association between Third Mobile Window Disorders and Symptom Reduction Using a Noise Cancelling Device: Inverse Tullio Phenomena

**DOI:** 10.3390/audiolres13040046

**Published:** 2023-07-17

**Authors:** Debby Feinberg, Mark Rosner, Gerard Gianoli

**Affiliations:** 1 NeuroVisual Medicine Institute, Bloomfield Hills, MI 48302, USA; drdebby@nvminstitute.org; 2 Ear and Balance Institute, Covington, LA 70433, USA; ggianoli79@gmail.com

**Keywords:** Tullio phenomena, noise cancellation device, dizziness, vertical heterophoria, third mobile window disorder, binocular vision dysfunction

## Abstract

Identifying a vestibular source of pathology in patients complaining of post-traumatic brain injury (TBI) dizziness can be difficult. We describe a possible new method utilizing a reduction in post-TBI symptoms (including dizziness) with the use of a noise cancellation device (NCD). This retrospective case series included patients with TBI and dizziness presenting to a binocular vision specialty clinic, who were diagnosed with a vertical heterophoria (VH). If they did not respond adequately to microprism lenses and/or if they experienced hyperacusis, they were evaluated with an NCD. If there was marked reduction in TBI symptoms (including dizziness), the patients were referred to a neuro-otologist for vestibular diagnostic evaluation and treatment. Fourteen patients were identified and found to have abnormalities on vestibular testing consistent with third mobile window disorder (TMWD). All were treated with a 6-week medical protocol (diuretics, no straining, low sodium/no caffeine diet). Five responded positively, requiring no further treatment. Nine required surgical intervention and responded positively. In conclusion, in 14 patients with post-concussive dizziness and VH, a positive response to NCD was associated with abnormal vestibular testing, a diagnosis of TMWD, and symptom reduction/resolution with a medical or surgical approach. The removal of sound resulting in reduction or resolution of vestibular symptoms represents an inverse Tullio phenomenon.

## 1. Introduction

Dizziness is a symptom with a multitude of causations, including pathology of vestibular, visual, cardiovascular, and/or neurologic origin. Among the visual causations for dizziness is vertical heterophoria. Vertical heterophoria (VH) is a form of binocular vision dysfunction (BVD) where the line of sight from one eye is slightly above the line of sight from the other eye when visual fusion is disrupted. The etiology of this misalignment most commonly emanates from either the visual system (superior oblique palsy) or vestibular system via the vestibular ocular reflex (the leading suspect under consideration is utricular dysfunction). VH treated with microprism lenses affords significant improvement in the symptoms of dizziness [1,2,3].

In 2016, one of the authors observed in their clinical practice that in those with VH who had residual dizziness despite treatment with microprism lenses, hyperacusis was frequently experienced, and that utilization of noise cancelling devices (NCDs) led to a further reduction in dizziness, improvement in balance and gait stability [4]. Additionally, an improved ability to obtain an accurate binocular alignment vision prescription (accomplished with microprism lenses) was afforded to patients using NCDs. The response to the NCDs was suspected to be a variation on the manifestation of Tullio phenomena [5]. Consequently, neurotology consultation and vestibular testing was performed in this subset of patients. Identification of a vestibular source for dizziness in these VH patients was observed in many, forming the basis for the current study [6]. The purpose of this paper is to describe an association between the positive response (i.e., a reduction in symptoms) to NCDs in a group of traumatic brain injury (TBI) patients with dizziness presenting to a binocular vision specialty clinic that had been diagnosed with VH and a vestibular disorder known as third mobile window disorder or TMWD. TMWD encompasses a group of disorders that have in common a discontinuity of the normally sealed inner ear. The two most common types of TMWD are bony dehiscences and perilymph fistulas. We will detail the presenting symptoms, NCD trial outcomes, vestibular test results, vestibular diagnoses, and treatment outcomes in this unique group of post-TBI patients with VH and dizziness.

## 2. Materials and Methods

**Figure 1:**
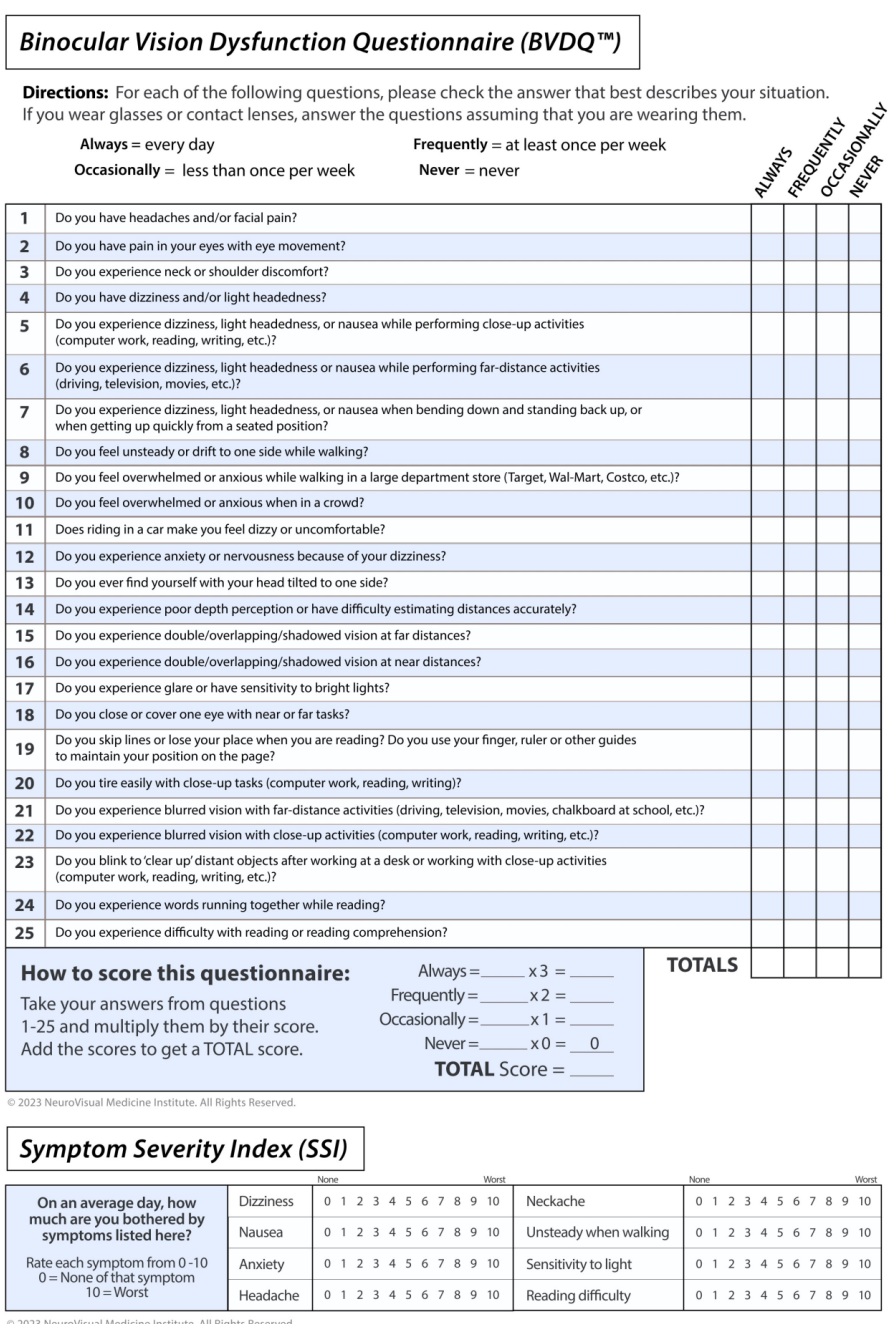
Binocular Vision Dysfunction Questionnaire (BVDQ) score and Symptom Severity Index (SSI).

This retrospective case series from 11/2011 to 11/2019 involves 14 TBI patients assessed for dizziness at a binocular vision specialty clinic. Patients who had persistent dizziness despite appropriate treatment with microprism lenses were evaluated with an NCD. Those who experienced a marked reduction in symptoms with the NCD were sent to a neuro-otologist for consultation and vestibular testing. Baseline data collected included demographics, Dizziness Handicap Inventory (DHI) score, Binocular Vision Dysfunction Questionnaire (BVDQ) score, Symptom Severity Index (SSI) total and subtest scores, top two chief complaints at presentation and their duration, presence of hyperacusis, and history of TBI. The BVDQ is a validated survey instrument containing 25 questions that is utilized to screen for binocular vision dysfunction (BVD) (Figure 1). The SSI is the sum of eight questions that assess the severity of the major symptoms of BVD on a 0–10 scale, in which dizziness and gait stability are included (Figure 1). The SSI was used to assess the effect the NCD had upon the patients’ set of symptoms during the binocular vision evaluation.

The binocular vision specialist performed a complete eye examination and, in addition, a detailed neuro-visual evaluation. This evaluation includes Maddox rod testing and red lens testing in multiple positions of gaze, as well as gait, posture, and balance analysis. A reduction in symptoms with the use of microprism lenses establishes the diagnosis of BVD and identifies the treatment as well.

If the patient did not experience significant symptom reduction with microprism, upon return evaluation, NCDs (Bose QC25 headphones, Bose Corporation, Framingham, MA, USA) were placed upon the patient during the assessment, and if a positive response was obtained, they were included as part of the patient’s treatment. A positive response was defined as either an immediate reduction in the SSI score and/or improvement in gait stability. Positive responses to the NCD resulted in referral for neuro-otologic evaluation.

The neuro-otology consultation included audiologic and vestibular testing consisting of comprehensive audiometry, impedance testing, cervical vestibular-evoked myogenic potential (cVEMP) testing, electrocochleography (ECOG), computerized platform posturography, platform pressure testing, rotational chair testing, and video electronystagmography (ENG) analysis. Additionally, all patients underwent an MRI of brain/IAC’s (internal auditory canals) and a 0.12 mm thin slice CT of the temporal bones.

Video ENG analysis included caloric testing, but also included Tullio, fistula, nasal Valsalva, and glottic Valsalva testing. These tests are described here. All had a baseline recording prior to testing and during testing, as well as a post-test recording using video-oculography and patient symptoms as outputs.

Tullio testing was performed with a portable audiometer presenting a pulsing 500 Hz tone at 105 dB to a single ear while recording the patient’s eye movement and concomitant symptoms. A normal response was no nystagmus and no symptoms elicited. An abnormal response was elicited nystagmus and concomitant symptoms of motion/dizziness. A suspect abnormal result was either symptoms but no nystagmus or nystagmus but no symptoms.

Fistula testing was performed with alternating positive and negative pressure applied by the physician with a Bruening otoscope under direct visualization of the tympanic membrane while video recording eye movements. An abnormal result was (1) phase-locked eye movement with direction changing with positive or negative pressure application and the patient experiencing symptoms of shifting/rocking, or (2) nystagmus and the patient experiencing rotary vertigo. A suspect abnormal result was the presence of eye movement without symptoms or the presence of symptoms without eye movement. The normal result was no eye movement and no symptoms.

For nasal Valsalva testing, the patient was instructed to inhale deeply and then hold their nose while continuously insufflating the middle ears for 25 s. A normal result was no nystagmus and no symptoms. An abnormal result was nystagmus (or change in nystagmus) associated with symptoms of dizziness/vertigo. A suspect abnormal result was either nystagmus (or change in nystagmus) or symptoms of dizziness/vertigo.

Glottic Valsalva testing was accomplished similar to nasal Valsalva testing, except the patient was instructed to strain under a closed glottis. Normal, abnormal, and suspect abnormal results were defined as for nasal Valsalva testing.

After audio-vestibular testing, the patients underwent medical and/or surgical treatment and vestibular rehabilitation. Medical therapy typically entailed acetazolamide therapy, sodium/caffeine restrictions, strain avoidance, and vestibular rehabilitation exercises. Outcome from treatment was also recorded. The surgical procedures deployed included superior canal resurfacing, posterior canal resurfacing, round/oval window reinforcement, endolymphatic sac decompression, and, in one case, reinforcement of the area above the oval window where the facial nerve created a dehiscence at the horizontal semicircular canal. The above data were recorded, and descriptive statistical analysis was performed.

Patients were diagnosed with TMWS if (1) symptoms were consistent with TMWS, (2) at least two positive objective tests corroborating these symptoms were obtained, and (3) CT scan identified a dehiscence. In the cases where no dehiscence was identified, they were labeled “perilymph fistula” (PLF). A potentially more accurate description of this group might be “CT Negative TMWS”, as this group most likely includes those with (1) near dehiscence, (2) a dehiscence that is yet to be identified, or (3) an intermittent round or oval window PLF.

The procedures followed were in accordance with the ethical standards of the responsible committee on human experimentation and with the Helsinki Declaration. The Salus Institutional Review Board approved this study.

## 3. Results

**Figure 2:**
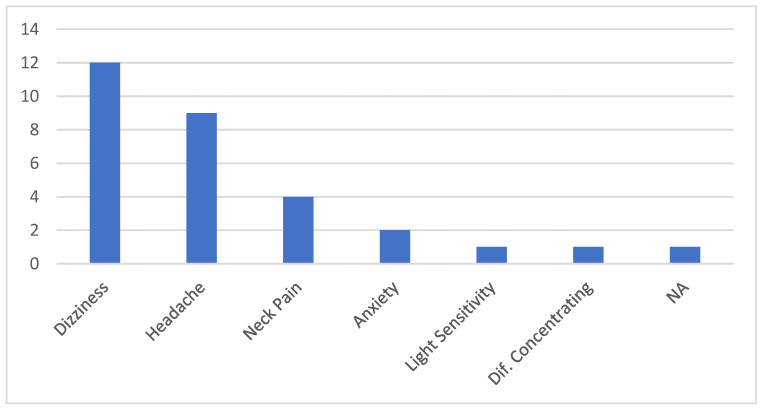
Two worst symptoms for each patient upon presentation to the binocular vision specialist.

Fourteen patients were included in this study. The average age was 46 (18–64) and there was a female preponderance (86%). Only 8 (57%) of the 14 had prior awareness of sound sensitivity or hyperacusis. The patients reported their top two symptoms as dizziness (12/14, 86%) and headache (8/14, 57%). (Figure 2) The duration from symptom onset to evaluation ranged from 1 year to 17 years, with a mean of 6.4 years.

**Figure 3:**
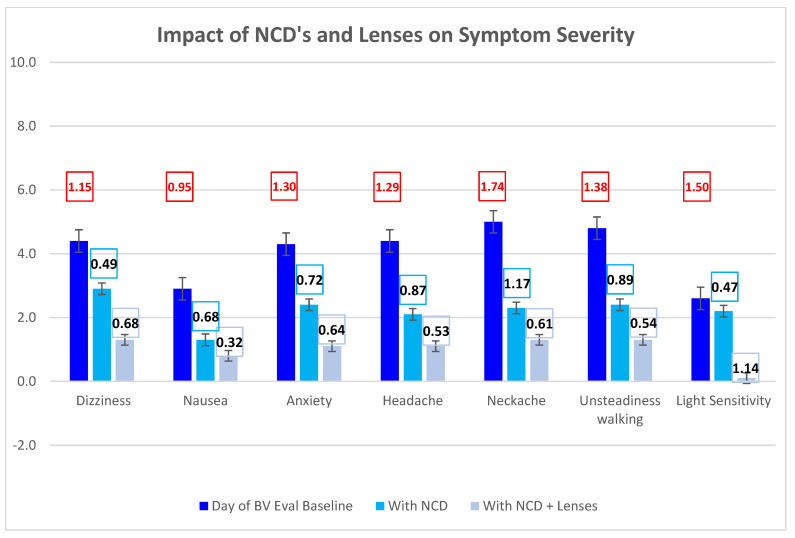
Symptom Severity Index (SSI) component scores during binocular vision exam at baseline, with trial of NCD, and with both NCD and microprism lenses. Cohen’s d ranged from 0.47 to 1.17 with NCD trial, from 0.32 to 1.14 with the addition of lenses, and from 0.95 to 1.74 from initial to final scores (in red).

Use of the NCD and microprism lenses by the optometrist during binocular vision evaluation resulted in a marked reduction in symptoms. Cohen’s d ranged from 0.47 to 1.17 with the NCD trial, from 0.32 to 1.14 with the addition of lenses, and from 0.95 to 1.74 from baseline to final values when wearing both NCDs and lenses (Figure 3). All patients were diagnosed and treated for either superior oblique palsy (SOP) or VH. SOP was treated with 0.25D to 1.00D prism (mean 0.46D); VH was treated with 0.5D to 4.00D divided between both eyes (mean 1.62D). One patient was additionally diagnosed with exophoria.

**Figure 4:**
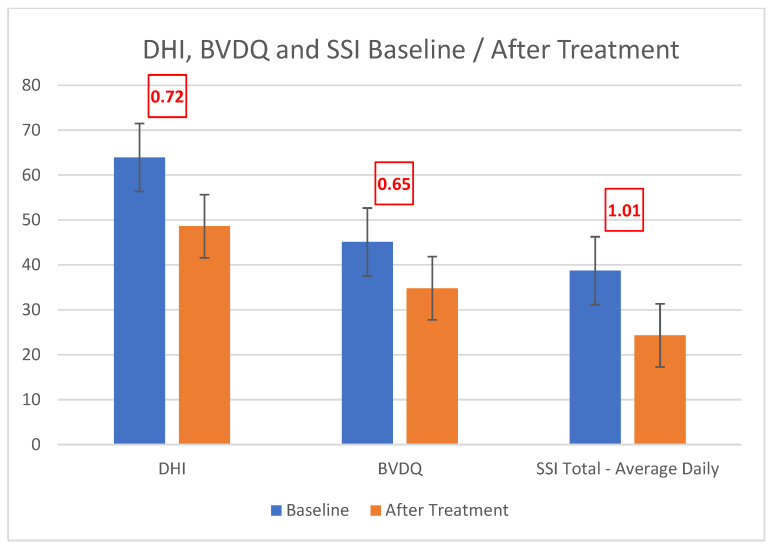
Decrease in DHI (Cohen’s d = 0.72), BVDQ (Cohen’s d = 0.65), and SSI (Cohen’s d = 1.01) scores after treatment of VH with microprism and treatment of vestibular disorders with medication/surgery.

**Figure 5:**
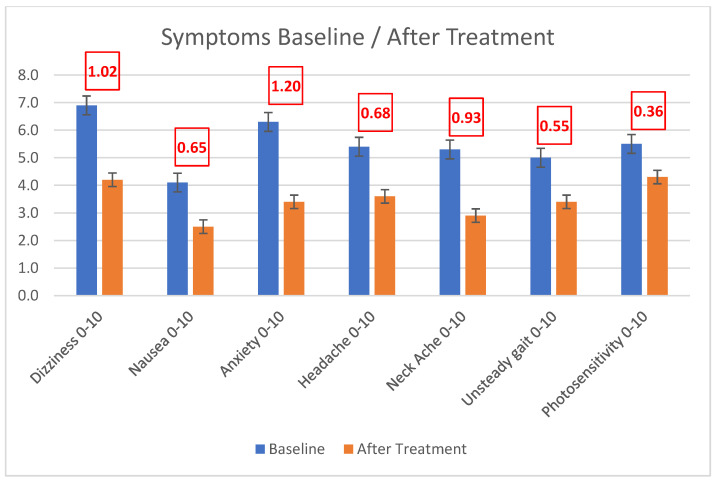
Reduction in Symptom Severity Index (SSI) component scores after treatment of VH with microprism and treatment of TMWS with medication/surgery, from photosensitivity (Cohen’s d = 0.36) to anxiety (Cohen’s d = 1.20).

Treatment with spectacle lenses (with correction for hyperopia, myopia, astigmatism, and heterophoria) as well as medical/surgical management of the TMWD resulted in a significant reduction in the scores of the instruments validated to assess dizziness (DHI) and binocular vision dysfunction (BVDQ) (Cohen’s d 0.72 and 0.65, respectively, as in Figure 4), as well as the associated symptoms (Cohen’s d ranging from 0.36, i.e., photosensitivity, to 1.20, i.e., anxiety) (Figure 5). When queried about improvement due to treatment (subjective % improved), patients reported an average 60.4% improvement.

The audiovestibular evaluation summary can be seen in Table 1.

Among 27 ears (14 patients), audiometric evaluation demonstrated normal hearing in 17 ears, sensorineural hearing loss in 8 ears, conductive hearing loss in 1 ear, and mixed hearing loss in 1 ear. Caloric testing revealed 5 with balanced findings, 5 with unilateral loss, 2 with bilateral loss, and 2 with a significant directional preponderance only. Sinusoidal harmonic acceleration (SHA) testing demonstrated findings of decreased gain in 8 cases, normal gain in 1 case, and increased gain in 2 cases. In 3 cases, SHA was unable to be accomplished, usually due to weight constraints. In the two cases of bilateral reduced caloric response, both had severely reduced gain on SHA. Asymmetry was found in 8 cases on SHA.

ECOG was abnormal in 4 ears, and normal in 23 ears. cVEMP was accomplished in all 27 ears. One had no response (a case where there was also no caloric response in that ear), fifteen had thresholds of 105 dB, nine had thresholds at 85 dB, and two were at 75 dB.

Tullio testing was abnormal in 12 ears, suspect abnormal in 7 ears, and normal in 8 ears. Fistula testing was abnormal in 13 ears, suspect abnormal in 6 ears, and normal in 8 ears. Platform pressure testing was abnormal in 5 ears, suspect abnormal in 5 ears, and normal in 11 ears. Platform pressure test was unable to be accomplished in 6 ears due to inability to stand on SOT 5 or due to weight constraints. Nasal Valsalva testing was abnormal in 6 patients, suspect abnormal in 3 patients, and normal in 5 patients. Glottic Valsalva was abnormal in 5 patients, suspect abnormal in 4 patients, and normal in 5 patients. Overall, 13 of 14 patients had at least 2 of the above tests, resulting in abnormal findings suggestive of TMWD. In all 14 patients, MRI with gadolinium (Gd) contrast did not reveal an abnormality of the internal auditory canal, cerebellar pontine angle, or other lesions to explain their symptoms. High-resolution CT scan demonstrated abnormalities in 8 of the 14 patients. Abnormalities found on CT scan are listed in Table 2.

The ears (N = 27) were diagnosed with the following: 10 perilymph fistula (or CT-negative TMWD), 7 superior semicircular canal dehiscence, 3 cochlear–facial dehiscence, 3 posterior semicircular canal dehiscence, 2 enlarged vestibular aqueduct, and 2 horizontal semicircular canal-facial nerve dehiscence.

Five patients responded to medical treatment alone. Nine patients, although improved with medical therapy, required surgical intervention. All patients had significant improvement or complete resolution of their vestibular complaints and sound sensitivity.

Audiometrically, 7.5% (2) of ears had conductive gaps on pure tone testing with normal tympanometry and intact acoustic reflexes, 30% (8) had sensorineural hearing loss, and 4% (1) had a mixed hearing loss.

Patient specific information regarding testing results, diagnosis, treatment, and outcomes can be seen in Table 3.

## 4. Discussion

BVD has been identified to occur secondary to concussion [7]. Among the etiologies for BVD is vestibular pathology. Specifically, an asymmetric counter-rolling effect from abnormal utricular stimulation can cause BVD [8]. Treatment for BVD typically employs the use of microprism lenses to ameliorate visual misalignment [2]. The patients in this study were all post-concussive and all reported significant improvement with microprism lenses. They reported further improvement in their symptoms with the use of the Bose QC25 NCD headphones (Bose Corporation, Framingham, Massachusetts), documented by validated instruments (DHI, BVDQ) and subjective symptom assessment/scoring (SSI). As noted in Figure 3, the combination of microprism lenses and NCDs led to a further improvement in symptoms beyond the use of microprism lenses alone.

Noise-cancelling devices are active sound reduction devices aimed at reducing low frequency noise. While traditional headphones and earplugs are generally effective at reducing higher frequencies, they are much less effective when reducing lower frequency (≤1000 Hz) sound. Noise-cancelling devices employ microphones to measure incoming low frequency sound and have an active output of low frequency sound in the opposite phase (anti-phase) of the incoming sound. This results in the “cancellation” effect [9]. Theoretically, NCDs would significantly reduce both low and high frequency sound-induced vestibular stimulation in sound-sensitive patients who wear them. To the best of our knowledge, this is the first reported study noting the association between a positive response to NCDs and the presence of concomitant TMWD, as well as the first study using NCDs as a therapeutic measure for TMWD.

The Tullio phenomenon and hyperacusis have been reported in TMWD [10]. Head trauma has been implicated as a common “second event” causing the onset of TMWD in these patients [11]. This study looks at a specific group of patients who suffered concussions (mTBI) and developed the symptoms of BVD, including dizziness. Binocular vision evaluation demonstrated VH and dizziness that improved with NCD use. The majority of patients (8/14) were aware of hyperacusis as a symptom prior to NCD use, and use of microprism lenses improved their symptoms. Further improvement was accomplished using NCDs. This combination of symptoms (VH and dizziness) along with improvement using NCDs raised the question of TMWD for the source of these symptoms.

The Tullio phenomenon is provocation of vestibular stimulation by sound (represented by nystagmus and vertigo). Using NCDs, we have revealed the inverse of the Tullio phenomenon—removal of sound results in resolution (or great improvement) in vestibular symptoms. The remarkable feature in this study group is that four patients were completely unaware that sound was problematic until they were given a trial of NCD. It is likely that the sound stimulation was ever-present and, thus, unable to be noticed by these patients until it was withdrawn by using the NCDs.

Vestibular testing demonstrated abnormalities in all patients, with 64% having abnormal caloric testing and 73% of patients assessed with rotational chair testing experiencing decreased gain, including severe gain reduction in the two patients with bilateral caloric weakness.

Most notable was the results on the Tullio testing, VNG fistula testing, VNG glottic Valsalva testing, VNG nasal Valsalva testing, and platform pressure testing. Among the patients evaluated, 93% (13/14) had abnormal or suspect abnormal results on at least two of the above tests. Given the high incidence of abnormalities on these tests, it is not surprising to find so many otic capsule dehiscences (57%) among this cohort (Table 2).

cVEMP testing and ECOG testing were relatively less impressive in this patient population. Only 14% of ears demonstrated an ECOG abnormality. Only 33% of ears had a cVEMP threshold at 85dB, 7.5% had a cVEMP threshold at 75 dB, and none were below this level. In our lab, 85 dB is considered normal, but given that many of these patients also had concomitant caloric weakness, it raises the question as to whether these ears have had saccular damage in addition to their documented horizontal semicircular canal deficit. Saccular damage, presumably from their head injury or ongoing TMWD could have muted the response to cVEMP. Of note, the only patient who had no response on cVEMP was a patient who had prior surgery for a superior semicircular canal dehiscence but was still symptomatic from a posterior canal dehiscence in the same ear.

High-resolution CT demonstrated that 57% of patients had a variety of labyrinthine dehiscences, while 43% were diagnosed with perilymph fistula (or CT-negative TMWD). All patients improved with medical management. Furthermore, 36% have successfully remained on medical therapy alone, while 64% required additional symptomatic relieve and eventually underwent dehiscence repair or window reinforcement procedures with successful outcomes.

This study demonstrates the association of a positive response to NCDs to patients being identified with TMWD in a cohort who had post-concussive BVD. The duration of dizziness from time of concussion until evaluation ranged from 1–17 years, with a mean of 6.4 years. This group of post-TBI patients had been through multiple other treatments without success and were considered chronic. What is most interesting is that 29% were not aware of any sound sensitivity prior to NCD use. This implies that there are patients who have sound-induced vestibular stimulation and do not recognize sound as a provocateur. Furthermore, NCDs were also useful as a treatment modality for this patient population. All the patients had symptom reduction using NCDs. Among the patients successfully treated with medical therapy, some still utilize the NCDs.

The placebo effect should always be considered. While there is no placebo-controlled group for this study, the immediate onset of relief and the immediate cessation of relief with placement and removal of the NCDs in this patient population mitigates the argument in favor of a placebo effect.

Additionally, the patients continue to wear the NCD for weeks/months and obtain benefit, which disappears immediately upon removal of the NCD. This is not a pattern consistent with a placebo effect. Lastly, as the patients received treatment with diuretics and/or surgical resolution of their TMWS, the need for the NCD was resolved. Regarding psychological problems in this cohort, anxiety was a fairly prevalent symptom, but the other symptoms they exhibited were not psychologically based. Additionally, those symptoms (as well as the anxiety) improved markedly with appropriate diagnosis and treatment. Almost every patient had been told at some point in their search for symptomatic help that they were imagining their symptoms or were pursuing secondary gain—that their symptoms were psychological in nature—and yet their symptoms improved when the correct diagnoses were made and the correct treatments were applied.

A strength of this retrospective case series is that in addition to the baseline data, post-treatment data were also available. The weaknesses of this study is the presumption that in this cohort, VH and sound stimulation are mediated by abnormal utricular stimulation. Unfortunately, none of the vestibular tests looked at utricular function. The vestibular test abnormalities noted above can only imply utricular damage by association (i.e., caloric weakness implying diffuse labyrinthine damage and, hence, utricular damage). Our lab has since begun using oVEMP and subjective visual vertical and video ocular counter roll testing to assess utricular function. We have identified NCDs as a potential screening test for TMWD for this patient population. However, because we have no data on those patients who failed NCD screening, we cannot make any assessment as to sensitivity or specificity of NCD as a testing measure. This will require a prospective analysis.

## 5. Conclusions

A positive response to NCDs in a binocular vision specialty clinic was associated with a diagnosis of TMWD among a group of patients who had suffered concussions and were complaining of dizziness. This was confirmed with vestibular testing. Medical and/or surgical intervention proved helpful in this patient population. Additionally, the use of NCDs uncovered an inverse of the Tullio phenomenon, namely removal of sound resulting in an improvement/resolution in vestibular symptoms and vision misalignment.

## Figures and Tables

**Table 1:** Audiovestibular testing summary.

					
Audiometry	Normal	SNHL	Negative Bone Scores	CHL	MHL
N = 27 ears	16	8	0	2	1
					
cVEMP Threshold	105 dB	85 dB	75 dB	No Response	
N = 27 ears	15	9	2	1	
					
ECOG	Normal	Abnormal	No Response		
N = 27 ears	23	4	0		
					
Caloric Testing	Normal	Unilateral Weakness	Bilateral Weakness	Directional Preponderance	
N = 14 patients	5	5	2	2	
					
SHA Gain	Normal	Reduced	Increased	Technically Limited	
N = 14 patients	1	8	2	3	
					
SHA Asymmetry	Normal	Abnormal	NA		
N = 14 patients	2	8	4		
					
Platform Pressure Test	Normal	Abnormal	Suspect Abnormal	Technically Limited	
N = 27 ears	11	5	5	6	
					
Tullio Test	Normal	Abnormal	Suspect Abnormal		
N = 27 ears	8	12	7		
					
Fistula Test	Normal	Abnormal	Suspect Abnormal		
N = 27 ears	8	13	6		
					
Nasal Valsalva	Normal	Abnormal	Suspect Abnormal		
N = 14 patients	5	6	3		
					
Glottic Valsalva	Normal	Abnormal	Suspect Abnormal		
N = 14 patients	5	5	4		

**Table 2:** CT Findings (N = 8).

2 Patients	Bilateral SSCD
1 Patient	Bilateral SSCD and unilateral PSCD
1 Patient	Unilateral SSCD and bilateral CFD
1 Patient	Bilateral EVA
1 Patient	Bilateral PSCD
1 Patient	Bilateral HSC-FND
1 Patient	Unilateral CFD

SSCD = superior semicircular canal dehiscence; PSCD = posterior semicircular canal dehiscence; CFD = cochlear facial dehiscence; EVA = enlarged vestibular aqueduct; HSC-FND = horizontal semicircular canal–facial nerve dehiscence.

**Table 3:** Patient specific information.

Pt Age	Pt Sex	Patient—Ear	Sxs (Yrs)	Tx	Outcome	DHI	Dx	Audio	PPT	Tullio	FT	N. Valsalva	G. Valsalva	Caloric	c-VEMP	Chair Gain	Chair Asym	ECOG
38	M	#1—Right	6 Yrs	Surgical	Successful	34	SSCD	Normal	Neg	Pos	Neg	Pos	Neg	Bil (TES 16)	105	Reduced—severe	NA	0.72
		#1—Left		Surgical	Successful		SSCD	Unilateral 6k notch	Neg	Pos	Neg	Pos	Neg	Bil (TES 16)	85	Reduced—severe	NA	0.45
46	F	#2—Right	4 Yrs	Surgical	Successful	94	LVAS— bil (R > L)	Unilateral 6k notch	Neg	Pos	Neg	Pos	Pos	1%, DP 34%	105	Reduced	Right	Neg
		#2—Left		Surgical	Successful		LVAS— bil (R > L)	Normal	Neg	Pos	Pos	Pos	Pos	1%, DP 34%	105	Reduced	Right	Neg
41	F	#3—Right	17 Yrs	Medical	Successful	56	PLF	Bil flat snhl (30)	Neg	Neg	Pos	Pos	Neg	Bil (TES 14)	105	Reduced	Left	0.62
		#3—Left					PLF	Bil flat snhl (30)	Neg	Neg	Pos	Pos	Neg	Bil (TES 14)	105	Reduced	Left	0.18
23	F	#4—Right	13 Yrs	Surgical	Successful	76	SSCD/FCD	Normal	Pos	Pos	Pos	Neg	Neg	21%R	75	Normal	Right	Neg
		#4—Left		Surgical	Successful		FCD	Normal	Pos	Neg	Pos	Neg	Neg	21%R	75	Normal	Right	Neg
57	F	#5—Right	3 Yrs	Surgical	Successful	74	SSCD	Normal	TL	Neg	Neg	Neg	Neg	100% R	105	Reduced	Right-severe	Neg
		#5—Left		Surgical	Successful—cx—SNHL		SSCD	L-LF, CHL	TL	Neg	Neg	Neg	Neg	100% R	105	Reduced	Right-severe	Neg
52	M	#6—Right	8 Yrs	Surgical	Successful	82	PLF	Normal	Susp	Pos	Pos	Susp	Susp	Neg	105	Reduced	Susp Left	0.21
		#6—Left		Surgical	Successful		PLF	Normal	Pos	Pos	Neg	Susp	Susp	Neg	105	Reduced	Susp Left	0.13
63	F	#7—Right	7 Yrs	Medical	Successful	44	HSC-FND	Normal	TL	Neg	Pos	Pos	Pos	26%—R	105	Reduced	Right	0.18
		#7—Left					HSC-FND	Normal	TL	Neg	Neg	Pos	Pos	26%—R	85	Reduced	Right	0.29
49	F	#8—Right	15 Yrs	Medical	Successful	36	PLF	HF-SNHL asym.	TL	Pos	Pos	Pos	Pos	Neg	85	Reduced	Left	0.3
		#8—Left		Surgical	Improved after left ear surgery		PLF	HF-SNHL asym.	TL	Pos	Pos	Pos	Pos	Neg	85	Reduced	Left	0.39
17	F	#9—Right	3 Yrs	Medical	Successful	34	PSCD	Normal	Pos	Pos	Pos	Neg	Pos	24%—L	85	Increased	Right	0.27
		#9—Left					PSCD	Normal	Pos	Pos	Pos	Neg	Pos	24%—L	85	Increased	Right	0.14
40	F	#10—Right	2 Yrs	Medical	Successful	76	PLF	Normal	Neg	Susp	Susp	Pos	Pos	24%—R	105	CNT	CNT	0.22
		#10—Left					PLF	Normal	Susp	Susp	Neg	Pos	Pos	24%—R	85	CNT	CNT	0.33
57	F	#11—Right	3 Yrs	Surgical	Successful	68	PLF/TBI	Normal	Susp	Susp	Susp	Neg	Susp	14%—L	105	CNT	CNT	0.31
		#11—Left		Surgical	Successful		PLF/TBI	Normal	Susp	Susp	Susp	Neg	Susp	14%—L	105	CNT	CNT	0.4
59	F	#12—Right	3 Yrs	Medical	Improved but could not tolerate meds	82	PLF?	Flat SNHL	Neg	Susp	Susp	Neg	Neg	2%—L	85	CNT	CNT	0.25
		#12—Left			No further follow up since		PLF	Flat SNHL	Neg	Pos	Pos	Neg	Neg	2%—L	105	CNT	CNT	0.15
40	F	#13—Right	1 yr	Surgical	Successful	76	FCD?	Normal	Susp	Susp	Susp	Susp	Susp	10%—R 40%—DP	85	Elevated	None	Neg
		#13—Left (no diagnosis)		None														
61	F	#14—Right	4 Yrs	Surgical	Successful	62	SSCD/PSCD	LF-MHL	Neg	Neg	Pos	Susp	Susp	11%—R	NR	Reduced	Right	Neg
		#14—Left		Medical	Successful		SSCD	Normal + A2IA1:I29 + A6:I29	Neg	Susp	Susp	Susp	Susp	11%—R	105	Reduced	Right	Neg

## Data Availability

The data presented in this study are available on request from the corresponding author. The data are not publicly available due to privacy.

## References

[1] Doble J.E., Feinberg D.L., Rosner M.S., Rosner A.J. (2010). Identification of binocular vision dysfunction (vertical heterophoria) in traumatic brain injury patients and effects of individualized prismatic spectacle lenses in the treatment of postconcussive symptoms: A retrospective analysis. PM&R.

[2] Rosner M.S., Feinberg D.L., Doble J.E., Rosner A.J. (2016). Treatment of vertical heterophoria ameliorates persistent post-concussive symptoms: A retrospective analysis utilizing a multi-faceted assessment battery. Brain Inj..

[3] Feinberg D.L., Rosner M.S., Rosner A.J. (2020). Vertical heterophoria treatment ameliorates headache, dizziness and anxiety. Opt. Vis. Perf..

[4] Feinberg D.L., Gianoli G.G. (2021). Personal communication.

[5] Lehmkuhl B., Andaloro C. (2022). Tullio Phenomenon. StatPearls [Internet].

[6] Gianoli G.G., Feinberg D.L. (2021). Personal communication.

[7] Zasler N.D., Katz D.I., Zafonte R.D. (2020). Brain Injury Medicine: Principles and Practice.

[8] Goto F., Ban Y., Tsutumi T. (2011). Acute audiovestibular deficit with complete ocular tilt reaction and absent VEMPs. Eur. Arch. Otorhinolaryngol..

[9] Gordon R.T., Vining W.D. (1992). Active noise control: A review of the field. Am. Ind. Hyg. Assoc. J..

[10] Ward B.K., Carey J.P., Minor L.B. (2017). Superior Canal Dehiscence Syndrome: Lessons from the First 20 Years. Front. Neurol..

[11] McCrary H.C., Babajanian E., Patel N., Yang S., Kircher M., Carlson M.L., Gurgel R.K. (2021). Superior Semicircular Canal Dehiscence Syndrome Following Head Trauma: A Multi-institutional Review. Laryngoscope.

